# Structural Analysis of an l-Cysteine Desulfurase from an Ssp DNA Phosphorothioation System

**DOI:** 10.1128/mBio.00488-20

**Published:** 2020-04-28

**Authors:** Liqiong Liu, Susu Jiang, Mai Xing, Chao Chen, Chongde Lai, Na Li, Guangfeng Liu, Dan Wu, Haiyan Gao, Liang Hong, Pan Tan, Shi Chen, Zixin Deng, Geng Wu, Lianrong Wang

**Affiliations:** a State Key Laboratory of Microbial Metabolism, School of Life Sciences and Biotechnology, Joint International Research Laboratory of Metabolic & Developmental Sciences, Shanghai Jiao Tong University, Shanghai, China; b Taihe Hospital, Hubei University of Medicine, Shiyan, Hubei, China; c Key Laboratory of Combinatorial Biosynthesis and Drug Discovery, Ministry of Education, School of Pharmaceutical Sciences, Wuhan University, Wuhan, China; d Department of Neurosurgery, Zhongnan Hospital, Wuhan University, Wuhan, China; e Jiangxi Engineering Laboratory for the Development and Utilization of Agricultural Microbial Resources, College of Bioscience and Bioengineering, Jiangxi Agricultural University, Nanchang, China; f National Facility for Protein Science Shanghai, Zhangjiang Lab, Shanghai, China; g School of Physics and Astronomy, Shanghai Jiao Tong University, Shanghai, China; University of Michigan-Ann Arbor

**Keywords:** DNA PT modification, Ssp system, cysteine desulfurase, crystal structure

## Abstract

DNA phosphorothioate (PT) modification, in which the nonbridging oxygen in the sugar-phosphate backbone is substituted by sulfur, is catalyzed by DndABCDE or SspABCD in a double-stranded or single-stranded manner, respectively. In Dnd and Ssp systems, mobilization of sulfur in PT formation starts with the activation of the sulfur atom of cysteine catalyzed by the DndA and SspA cysteine desulfurases, respectively. Despite playing the same biochemical role, SspA cannot be functionally replaced by DndA, indicating its unique physiological properties. In this study, we solved the crystal structure of Vibrio cyclitrophicus SspA in complex with its natural substrate, cysteine, and cofactor, pyridoxal phosphate (PLP), at a resolution of 1.80 Å. Our solved structure revealed the molecular mechanism that SspA employs to recognize its cysteine substrate and PLP cofactor, suggesting a common binding mode shared by cysteine desulfurases. In addition, although the distance between the catalytic Cys314 and the substrate cysteine is 8.9 Å, which is too far for direct interaction, our structural modeling and biochemical analysis revealed a conformational change in the active site region toward the cysteine substrate to move them close to each other to facilitate the nucleophilic attack. Finally, the pulldown analysis showed that SspA could form a complex with SspD, an ATP pyrophosphatase, suggesting that SspD might potentially accept the activated sulfur atom directly from SspA, providing further insights into the biochemical pathway of Ssp-mediated PT modification.

## Abstract (precis)

Apart from its roles in Fe-S cluster assembly, tRNA thiolation, and sulfur-containing cofactor biosynthesis, cysteine desulfurase serves as a sulfur donor in the DNA PT modification, in which a sulfur atom substitutes a nonbridging oxygen in the DNA phosphodiester backbone. The initial sulfur mobilization from l-cysteine is catalyzed by the SspA cysteine desulfurase in the SspABCD-mediated DNA PT modification system. By determining the crystal structure of SspA, the study presents the molecular mechanism that SspA employs to recognize its cysteine substrate and PLP cofactor. To overcome the long distance (8.9 Å) between the catalytic Cys314 and the cysteine substrate, a conformational change occurs to bring Cys314 to the vicinity of the substrate, allowing for nucleophilic attack.

## INTRODUCTION

Cysteine desulfurase, a pyridoxal phosphate (PLP)-dependent homodimer, strips sulfur from the l-cysteine substrate to generate l-alanine and a protein-bound persulfide intermediate on the active site cysteine residue. The persulfide sulfur is subsequently incorporated into a variety of sulfur-containing biofactors, such as tRNA thionucleotides, biotin, molybdopterin, lipoic acid, and iron-sulfur (Fe-S) clusters, which are essential for biosynthetic processes (1, 2). Cysteine desulfurase activity was first characterized for the NifS protein from the nitrogen fixation (NIF) system of Azotobacter vinelandii and later found in sulfur formation (SUF) and iron-sulfur cluster assembly (ISC) machineries as the initial stage of sulfur trafficking catalyzed by the paralogous cysteine desulfurases, SufS (also known as CsdB) and IscS, respectively (3–5).

As a versatile sulfur donor, cysteine desulfurase activity is also essential to DNA phosphorothioate (PT) modification, in which the nonbridging oxygen atom in the DNA sugar-phosphate backbone is replaced by sulfur (6, 7). The “writing” of a PT modification into DNA occurs in a sequence-selective and *R*_P_ configuration-specific manner governed by Dnd or Ssp machineries in bacteria and archaea (6, 8, 9). Dnd modification systems consist of five proteins (DndA, B, C, D, and E) and confer PTs in 4-bp complementary motifs, e.g., 5′-G_PS_AAC-3′/5′-G_PS_TTC-3′ (PS, phosphate-sulfur linkage) in Escherichia coli B7A and 5′-G_PS_ATC-3′/5′-G_PS_ATC-3′ in Hahella chejuensis KCTC2396 (10, 11). DndA is a cysteine desulfurase and can be functionally substituted by IscS *in vivo*, agreeing well with the observation that some bacterial genomes possess clustered *dndBCDE* but not *dndA* (12, 13). The catalytic cysteine in DndA undergoes nucleophilic reaction with the sulfur atom of its cysteine substrate to form an activated persulfide and then transfers the sulfur to the [4Fe-4S] cluster of the DndC protein (13, 14). DndC exhibits ATP pyrophosphatase activity and shows significant sequence homology to phosphoadenosine phosphosulfate reductase (13). DndB functions as a transcriptional regulator capable of sensing cellular ATP levels to control the transcription of the *dndBCDE* cluster (15, 16). DndD has ATPase activity and has been proposed to provide energy during sulfur incorporation (17). DndE adopts a tetramer conformation and displays preferred binding affinity for nicked double-stranded (ds) DNA *in vitro* (18). Owing to the nuclease tolerance of PT linkage, DndABCDE coupled to DndFGH constitutes a defense barrier analogous to a methylation-based restriction-modification system, i.e., DndFGH restricts non-PT-modified invading DNA (19).

Interestingly, we recently characterized a new type of Ssp system that exhibits different genetic organization, biochemical functions, and phenotypic behavior from Dnd systems. In contrast to the typical dsDNA PT modification mediated by Dnd systems, Ssp systems confer single-stranded (ss), high-frequency DNA PT modification (9). For instance, SspABCD confers ssDNA PT modification to 3-bp consensus sequences, e.g., 5′-C_PS_CA-3′ in Vibrio cyclitrophicus FF75, and the PT levels are 3- to 10-fold higher than those in DndBCDE-expressing E. coli B7A and *H. chejuensis* KCTC2396 (10, 20). In contrast to the simple self-nonself discrimination mechanism in the Dnd system, SspABCD functions together with SspE to provide protection against phage invasion in an unusual PT-dependent manner (9). In parallel with the companion ssDNA PT modification, SspE exerts its toxicity by introducing nicking damage on phage DNA and consequently impairs phage replication and disturbs phage propagation (9). Additionally, the redox and nucleophilic properties of PT sulfur render the PT modification a versatile player in maintenance of cellular redox homeostasis, epigenetic regulation, and environmental fitness (21, 22).

We have recently determined the crystal structures of SspB and SspE in the Ssp systems, elucidating the essential role of SspB as a nickase in the ssDNA PT formation and the dual functions of SspE as a PT-stimulated nucleoside triphosphatase (NTPase) and a nicking endonuclease in phage resistance (9). To provide further insights into Ssp-mediated PT modification, we determined the crystal structure of SspA from *V. cyclitrophicus* FF75 at a 1.80-Å resolution. Our biochemical and structural studies provide the molecular details of how SspA recognizes the cysteine substrate and the PLP cofactor. Moreover, modeling and experimental data show that SspA undergoes a conformational change to move the catalytic Cys314 near the cysteine substrate to facilitate sulfur transfer.

## RESULTS AND DISCUSSION

### Determination of the SspA structure.

**FIG 1:**
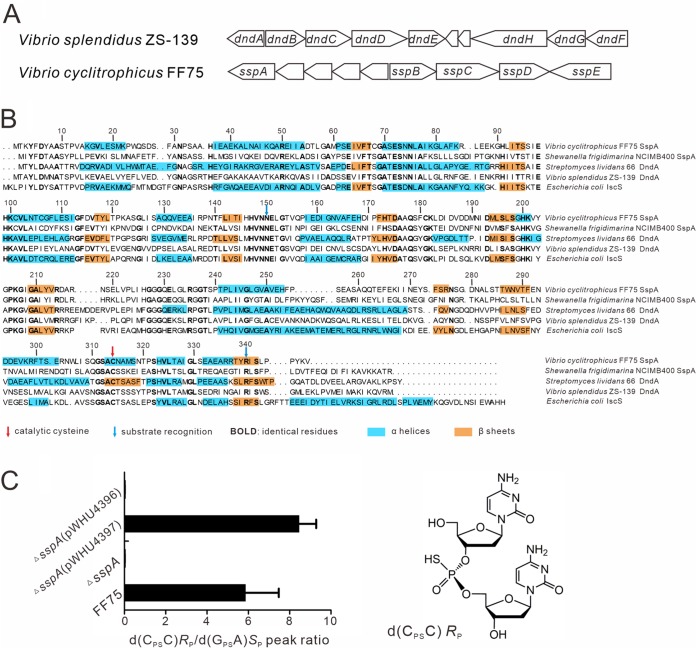
(A) A typical *dndABCDE-dndFGH* system in V. splendidus ZS-139 and *sspABCDE-sspE* in *V. cyclitrophicus* FF75 are displayed. (B) Structure-based sequence alignment of SspA, DndA, and IscS proteins. The catalytic cysteines are marked by a red arrow. Residues critical for recognizing substrate cysteines are indicated by blue arrows. Residues identical in all five sequences are shown in bold. α-Helices and β-sheets are shaded in cyan and yellow, respectively. (C) Detection of PT-linked, *R*_P_ stereospecific d(C_PS_C) dinucleotides in wild-type FF75 and mutants by LC-MS/MS. pWHU4396 and pWHU4397 plasmids were constructed to express DndA from V. splendidus ZS-139 and SspA from *V. cyclitrophicus* FF75, respectively. Chemically synthesized d(G_PS_A) in *S*_P_ configuration (5 pmol) was used as the reference. Data are representative of three independent experiments. PT-linked d(C_PS_C) dinucleotides in *R*_P_ configuration are shown in the structural inset.

SspA in the Ssp PT system of *V. cyclitrophicus* FF75 shares 56% and 57% sequence similarity with DndA in Vibrio splendidus ZS-139 and IscS in E. coli, respectively (Fig. 1A and B). However, the *sspA* mutation abolished DNA PT modification in *V. cyclitrophicus* FF75 despite the presence of the chromosomal *iscS* ortholog (Fig. 1C). Moreover, the cysteine desulfurase activity of SspA in *V. cyclitrophicus* FF75 cannot be functionally replaced by DndA from V. splendidus ZS-139 (Fig. 1C), prompting us to investigate the mechanism of sulfur transfer in the Ssp PT system. The catalytic cysteine, Cys314, of SspA, corresponding to Cys327 in Streptomyces lividans DndA and Cys328 in E. coli IscS, is responsible for nucleophilic attack on the cysteine substrate (14, 23). Indeed, the C314S point mutation in SspA abolishes d(C_PS_C) PT modification of *V. cyclitrophicus* FF75 (9). Here, we determined the crystal structure of the C314S mutant of SspA from *V. cyclitrophicus* FF75 in complex with its natural substrate, cysteine, at the resolution of 1.80 Å (see Table S1 in the supplemental material).

10.1128/mBio.00488-20.3 TABLE S1 Data collection and refinement statistics. Download Table S1, PDF file, 0.2 MB. Copyright © 2020 Liu et al. 2020 Liu et al. This content is distributed under the terms of the Creative Commons Attribution 4.0 International license.

**FIG 2:**
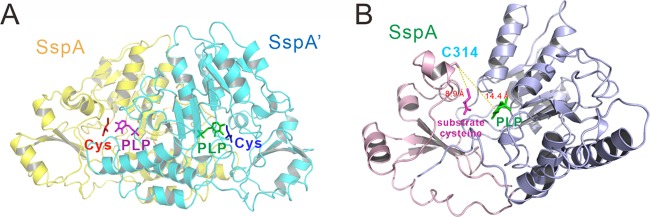
Crystal structure SspA from *V. cyclitrophicus* FF75. (A) Overall structure of the SspA dimer in complex with its cysteine substrate and PLP cofactor. The two protomers are shown in yellow and cyan. (B) Structure of a protomer of SspA. The larger N-terminal region (residues 1 to 254) and the smaller C-terminal region (residues 255 to 348) of SspA are colored in light blue and light pink, respectively. The PLP cofactor and cysteine substrate are colored in green and light magenta, respectively. The distance between the C314 catalytic cysteine and cysteine substrate and PLP cofactor is 8.9 Å and 14.4 Å, respectively.

In this structure, SspA forms a symmetric dimer (Fig. 2A), and its dimeric organization resembles that of other cysteine desulfurases, such as DndA and IscS. The fold of SspA is also similar to that of DndA (PDB code 3VAX) and IscS (PDB code 1P3W) with root mean square deviation (RMSD) values of 1.176 Å and 1.052 Å for 236 and 258 aligned Cα atoms, respectively (Fig. 1B). The SspA protein can be divided into two regions as follows: a larger N-terminal region (residues 1 to 254) possessing the PLP cofactor-binding site and a smaller C-terminal region (residues 255 to 348) bearing the Cys314 active site (Fig. 2B). The large region mostly consists of a seven-stranded parallel β-sheet flanked by seven α-helices and several tightly packed α-helices. The small region harbors a three-stranded antiparallel β-sheet flanked by four α-helices. The distance between Cys314 and the PLP cofactor is approximately 14.4 Å, and the distance between Cys314 and the cysteine substrate is 8.9 Å (Fig. 2B).

### Active site structure and the substrate-binding mechanism of SspA.

**FIG 3:**
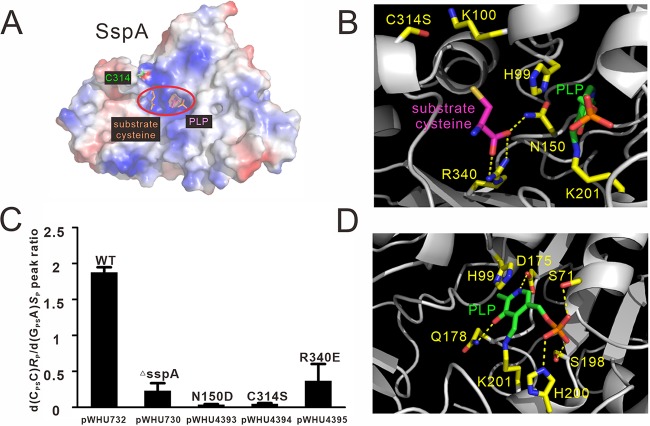
Crystal structure of SspA reveals residues critical to recognizing the cysteine substrate and PLP cofactor. (A) The cysteine substrate and PLP are located in a positively charged surface pocket (marked with a red oval) of SspA. (B) Polar interactions stabilizing the substrate cysteine in the active site are indicated by broken yellow lines. (C) LC-MS/MS detection of PT modification in E. coli Trans1-T1 harboring pWHU732 and derivatives. pWHU732 and pWHU730 plasmids express SspABCD and SspBCD from *V. cyclitrophicus* FF75, respectively. pWHU4393 to pWHU4395 plasmids were pWHU732 derivatives expressing SspBCD together with SspA_N150D_, SspA_C314S_, and SspA_R340E_. Chemically synthesized d(G_PS_A) (25 pmol) in the *S*_P_ configuration was used as the internal standard. Data are representative of three independent experiments. (D) PLP cofactor-binding interactions in SspA. The carbon atoms of PLP are colored in green. The carbon atoms of PLP-binding residues of SspA are colored in yellow. Nitrogen and oxygen atoms are colored in blue and red, respectively. SspA is colored in gray. Hydrogen bonds are displayed as yellow dashed lines.

In the structures of E. coli CsdB (PDB code 1C0N) and *Synechocystis* sp. strain PCC 6803 SufS (PDB code 1T3I), the catalytic cysteines are located on a short loop, while in S. lividans DndA (PDB code 3VAX), the catalytic cysteine resides on a β strand (Fig. S1). In contrast, the Cys314 active site cysteine in SspA occurs in a short α-helix (residues 313 to 318) with only six amino acid residues in length. This Cys314-resident helix is flanked by 9- (residues 304 to 312) and 4-amino-acid (residues 319 to 322) loops, which may allow versatile movement of Cys314 (Fig. S1). In terms of the cysteine substrate, it is located in a positively charged surface pocket in SspA (Fig. 3A). The side chain guanidinium group of Arg340 and the side chain amide group of Asn150 make three hydrogen bonds with the carboxyl group of the cysteine substrate (Fig. 3B). Similarly to C314S, R340E and N150D mutations in pWHU732, expressing *sspABCD* from *V. cyclitrophicus* FF75, remarkably impaired the PT modification in E. coli Trans1-T1 (Fig. 3C), which confirmed the essential roles of these residues in catalyzing the desulfurization reaction of the cysteine substrate. Moreover, both R340 and N150 are conserved in DndA and IscS (Fig. 1B), suggesting similar binding modes for cysteine substrates.

10.1128/mBio.00488-20.1 FIG S1 (A) In SspA, the Cys314 active site is located on an α-helix colored in cyan. This Cys314-resident helix is flanked by 9- (residues 304 to 312) and 4-amino-acid (residues 319 to 322) loops, which are colored in yellow. (B and C) In SufS (PDB code 1T3I) and CsdB (PDB code 1C0N), the active site cysteines, namely, Cys372 and Cys364, are located on loops. Cys372 and Cys364 are colored in yellow, and the loops are colored in pink and orange. (D) In DndA (PDB code 3VAX), the Cys327 active site is located on a β-strand. Cys327 is colored in yellow, and the β-strand is colored in magenta. Download FIG S1, TIF file, 2.7 MB. Copyright © 2020 Liu et al. 2020 Liu et al. This content is distributed under the terms of the Creative Commons Attribution 4.0 International license.

### Interaction interface between SspA and PLP.

The PLP cofactor is located between the larger and smaller regions and is spatially closer to the larger one (Fig. 2B). The PLP cofactor is covalently attached to the side chain amino group of Lys201 in a deep surface pocket via the formation of an internal aldimine Schiff base. Several additional polar and nonpolar interactions are involved in recognition of the PLP cofactor. The imidazole ring of His99 forms π-π stacking with the pyridine ring of PLP to make multiple van der Waals interactions. Additionally, at the bottom of the surface pocket, Asp175 forms two hydrogen bonds with the pyridine N1 atom of PLP. Gln178 from the bottom of the surface pocket forms a hydrogen bond with the hydroxyl group of the pyridine of the PLP. In addition, the phosphate group of PLP forms hydrogen bonds with the side chains of Ser71, Ser198, and His200 (Fig. 3D). These interactions are similar to those formed by PLP in the DndA and IscS structures, indicating a conserved molecular mechanism of PLP cofactor binding. Involvement of the multiple interactions ensures the fixation of PLP in the active site even when its internal Schiff base covalently bonded with Lys201 is broken in exchange of forming an external aldimine with the amino group of the cysteine substrate (23).

### Conformational change of the active site cysteine-containing helix in SspA.

Crystal structures of many cysteine desulfurases, including IscS, SufS, NifS, and DndA, as well as their complexes with interaction partners, such as the IscS-IscU, IscS-TusA, and CsdA-CsdE complexes, have been reported (14, 23–28). In the structures of these cysteine desulfurases, the conformations of loops containing the catalytic cysteines are highly variable, which is indicative of the exceptional flexibilities of these loops. The structures of the IscS-IscU and IscS-TusA complexes suggest that the conformational plasticity of the catalytic cysteine-harboring loop of IscS is essential for its ability to transfer sulfur to multiple acceptor proteins (26, 27). However, despite the vast number of structural and biochemical studies on cysteine desulfurases, the occurrence of such conformational change is unclear (14, 23–34).

**FIG 4:**
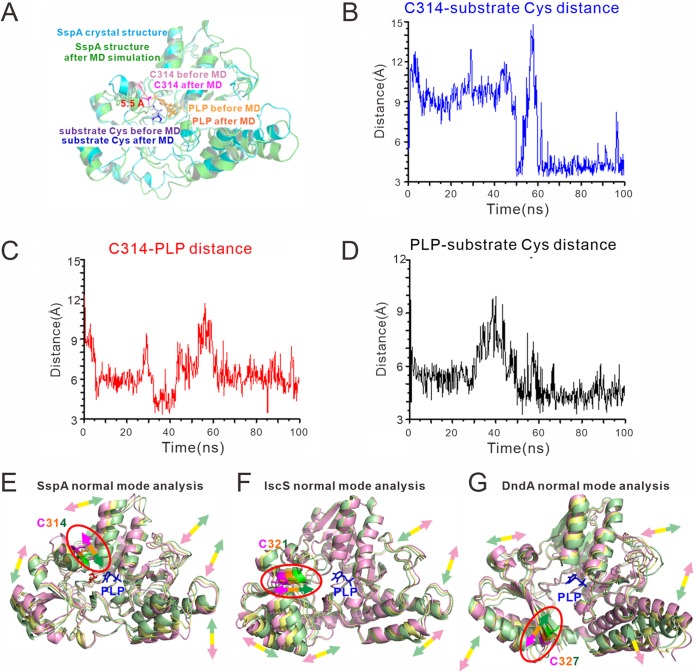
The catalytic cysteines of SspA and other cysteine desulfurases intrinsically move toward their substrate cysteines. (A) During molecular dynamics simulations, the C314 active site moved 5.5 Å toward the cysteine substrate. (B to D) Distances between C314 and the cysteine substrate (B), between C314 and PLP (C), and between PLP and the cysteine substrate (D) during the MD simulation. (E to G) Normal mode analysis shows that the C314 catalytic cysteine of SspA (E), C321 catalytic cysteine of IscS (F), and C327 catalytic cysteine of DndA (G) all intrinsically move toward the cysteine substrate/PLP.

In the structure of SspA, the distance between the Cys314 catalytic cysteine and the cysteine substrate is 8.9 Å, exceeding the upper limit for effective nucleophilic attack (Fig. 2B). This long distance has been observed in almost all cysteine desulfurases examined (14, 23–25, 30, 31, 33). Movement of the catalytic cysteine toward the substrate cysteine to enable nucleophilic attack might be inevitable. Therefore, we first performed a molecular dynamics (MD) simulation on the structure of SspA in complex with its substrate cysteine. After the MD simulation, the larger region of SspA exhibited relatively little structural difference with an RMSD value between the crystal structure and the MD-simulated structure being 1.617 Å for 207 aligned Cα atoms. In contrast, the smaller region of SspA underwent a relatively larger conformational change with an RMSD value of 1.820 Å for 79 aligned Cα atoms. In addition, there was a relative motion between the larger and smaller regions of SspA. The Cys314 catalytic cysteine moved 5.5 Å toward the cysteine substrate (Fig. 4A), shortening the distance between them to 3.4 Å (Fig. 4B), which is within the range of nucleophilic attack. The conformational change of the smaller region and its motion toward the larger region accounted mostly for the overall structural difference of SspA with an RMSD value of 2.051 Å for 285 aligned Cα atoms. The distance between Cys314 and PLP was also shortened accordingly (Fig. 4C), while the PLP-substrate cysteine distance did not substantially alter (Fig. 4D).

To corroborate the MD simulation results, we conducted a normal mode (NM) analysis, which is a convenient means to analyze intrinsic breathing motions and conformational changes of proteins (35, 36). NM analysis also revealed that the catalytic Cys314 had an intrinsic vibrational motion toward the cysteine substrate and the PLP cofactor (Fig. 4E). In various cysteine desulfurases, the catalytic cysteines reside in different locations and different secondary structure elements, such as α-helices (e.g., SspA), β-sheets (e.g., DndA), and loops (e.g., IscS). Nevertheless, no matter which secondary structure element the catalytic cysteine resides in, NM analysis indicated that it persistently exhibited intrinsic motion toward PLP and the putative substrate-binding site (Fig. 4F and G). Therefore, movement of the catalytic cysteine toward the substrate is an intrinsic property of cysteine desulfurases and independent of the relative locations or secondary elements where the catalytic cysteine is located.

**FIG 5:**
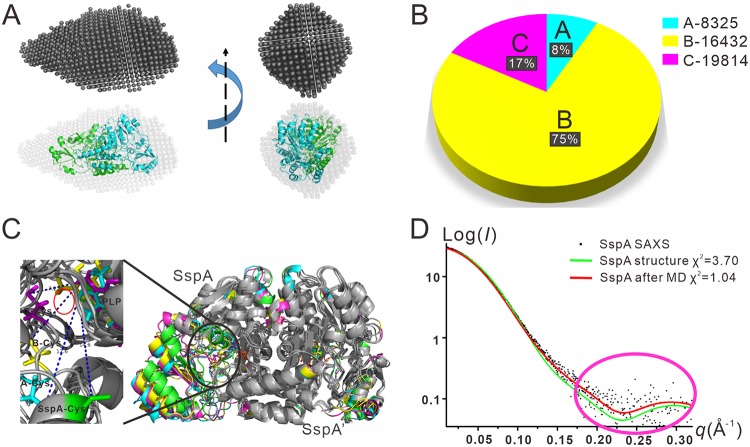
SAXS analysis of SspA reveals the intrinsic motion of the C314 active site of SspA toward its cysteine substrate. (A) An *ab initio* model of SspA was calculated using SAXS scattering data. The SspA crystal structure is superimposed. (B) The percentages of 8,325 (A), 16,432 (B) and 19,814 (C) clustered structures are displayed in blue, yellow, and magenta, respectively. (C) Superposition comparison of SspA and the cysteine substrate before and after simulation. The crystal structure of SspA is green (A is blue, B is yellow, and C is magenta). The diagram on the left is the distance graph between the active site and the PLP cofactor, and the red circle indicates the location of the l-cysteine substrate. (D) Comparison of the experimental SAXS scattering profile of SspA (black dots) with theoretical scattering profiles calculated using the SspA crystal structure (green line). The result of the cluster after molecular dynamics simulation was calculated according to the Monte Carlo method (red line).

To experimentally corroborate our results of MD simulation and NM analysis, we exploited small-angle X-ray scattering (SAXS) in combination with an MD-based conformational search using the crystal structure of the SspA dimer. First, we collected synchrotron SAXS data of purified SspA protein in solution. The SAXS data were then averaged to generate an *ab initio* model of SspA (Fig. 5A), which agreed well with our crystal structure. Next, we performed MD simulation to generate 20,000 simulated trajectories of SspA in 1,000 ns. Three clusters of calculated structures, named A (8%), B (75%), and C (17%), were obtained from the 20,000 simulations using the Monte Carlo method (Fig. 5B) (37). The superposition comparison between the three clusters of structures and the crystal structure of SspA revealed that the conformation of the large region in each protomer of SspA barely changed (Fig. 5C). In contrast, a conformational change extending outward at C314 occurred in the small region of one SspA protomer. The short α-helix harboring the active site changed into a loop and moved toward the substrate (Fig. 5C). However, a similar conformational change was not observed in the other SspA protomer, SspA′, suggesting that the catalytic processes in the two protomers do not occur simultaneously.

We calculated theoretical scattering profiles from the crystal structure of SspA (green line in Fig. 5D) and from the weighted square of the simulated structure clusters (red line in Fig. 5D). In the low-*q* region (i.e., overall structure at the macroscopic level), the theoretical scattering profile calculated from the crystal structure of SspA and the experimental SAXS scattering data matched perfectly. However, in the high-*q* range (i.e., detailed fine structure at the microscopic level, magenta circle in Fig. 5D), the theoretical profile calculated from the crystal structure of SspA exhibited considerable deviation from the experimental data. In contrast, the theoretical scattering profile calculated from the MD-simulated structures of SspA (red line in Fig. 5D) displayed remarkable correction, especially at the high-*q* range, and agreed much more with the experimental SAXS data (χ^2^ = 1.04 for the theoretical curve generated from the MD result versus χ^2^ = 3.70 for that from the crystal structure). Because the MD-simulated structure takes the mobility of the catalytic cysteine-resident loop into account while the crystal structure does not, our SAXS studies provide supportive experimental evidence that the catalytic cysteine of SspA moves toward the cysteine substrate.

### SspA and SspD form a complex.

IscS interacts with downstream sulfur-acceptor proteins, such as IscU, and their associations expedite the transfer of the activated sulfur atom (38, 39). The conformational plasticity of the cysteine-containing loop is essential for IscS to transfer sulfur to multiple acceptor proteins, such as IscU and TusA (26, 27). In the Dnd PT modification system, DndA promotes the assembly of the 4Fe-4S cluster in DndC, presumably by providing an activated sulfur atom (40, 41). Therefore, we investigated whether SspA in the Ssp system associates with SspD, which harbors pyrophosphatase activity *in vitro* as DndC (9). Due to the insoluble expression of SspD from *V. cyclitrophicus* FF75, we expressed both SspA and SspD from Shewanella frigidimarina NCIMB400. Pulldown experiments clearly showed that His-tagged SspD formed a complex with untagged SspA (see Fig. S2 in the supplemental material). Therefore, the flexibility and mobility of the SspA active site cysteine-containing loop are expected not only to be essential for nucleophilic attack on the cysteine substrate but also to play an important role in transferring the activated sulfur atom to SspD and eventually incorporating sulfur into the DNA backbone with the assistance of other Ssp proteins.

10.1128/mBio.00488-20.2 FIG S2 Interaction of SspA and SspD proteins. E. coli BL21(DE3) expressing recombinant His-tagged SspD and SspA (with its His tag removed by thrombin) proteins were used for a Ni^2+^-NTA column *in vitro* pulldown experiment. His-SspD-before-loading fraction, His-SspD flowthrough fraction, SspA-before-loading fraction, SspA flowthrough fraction, and eluate from the Ni^2+^-NTA column were analyzed by SDS-PAGE with Coomassie blue staining. The molecular weight marker is shown in the left lane. Download FIG S2, TIF file, 2.9 MB. Copyright © 2020 Liu et al. 2020 Liu et al. This content is distributed under the terms of the Creative Commons Attribution 4.0 International license.

### Conclusions.

In the most recently characterized Ssp system, SspABCD confers single-stranded PT via the DNA-nicking activity of SspB (9). The sulfur atoms required for PT modification are extracted from cysteines by cysteine desulfurase. SspA exhibits PLP-dependent cysteine desulfurase activity and is predicted to initiate the delivery of sulfur for ssDNA PT formation (9). In this study, the crystal structure of SspA in complex with its cysteine substrate was determined. Considering the similar overall fold and the conservation of substrate-binding residues, it is conceivable that the cysteine-binding molecular mechanisms of IscS, DndA, and SufS resemble that of SspA. Given that SspA and SspD form a complex, it is likely that SspD functions as the intermediate sulfur acceptor, analogous to the sulfur transfer from DndA to DndC, before the sulfur can be further supplied to PT bonds. In combination with the observations that both Ssp and Dnd systems employ cysteine desulfurases, i.e., SspA and DndA, as sulfur donors and their interactions with SspD and DndC pyrophosphatase, respectively, we find it plausible that Ssp- and Dnd-governed DNA PT modification systems may be derived from a common ancestor but that divergence occurred leading to distinct modes of DNA target selection. The structure showed that the distance between the Cys314 active site and the cysteine substrate is too great to allow Cys314 to form a persulfide bond with the PLP-bound cysteine substrate. Our study shows that cysteine desulfurases employ conformational changes to move the thiol group of their active site cysteines into the closer vicinity of their cysteine substrates to facilitate nucleophilic attack, thus providing a basis for further understanding of the molecular mechanism of sulfur transfer in PT systems.

## MATERIALS AND METHODS

### Bacterial strains and plasmids.

The bacterial strains and plasmids used in this study are listed in Table S2 in the supplemental material.

10.1128/mBio.00488-20.4 TABLE S2 Strains and plasmids used in this study. Download Table S2, PDF file, 0.3 MB. Copyright © 2020 Liu et al. 2020 Liu et al. This content is distributed under the terms of the Creative Commons Attribution 4.0 International license.

### PT detection by LC-MS/MS.

PT detection by liquid chromatography-tandem quadrupole mass spectrometry (LC-MS/MS) was conducted as previously described (20). Briefly, 20 μg of DNA was digested with nuclease P1 (US Biological, USA) followed by dephosphorylation by alkaline phosphatase (Sigma, USA). The enzymes were removed by ultrafiltration using a Nanosep 10K-column (Pall, USA). The filtrate, containing chemically synthesized d(G_PS_A) *S*_P_ as reference, was loaded onto a Thermo Hypersil Gold aQ column (150 × 2.1 mm, 3 μm). The column was coupled to a Thermo TSQ Quantum Access Max mass spectrometer for PT modification detection.

### Construction of pWHU730 and derivatives.

A 5,844-bp fragment harboring *sspBCD* was amplified from the genomic DNA of *V. cyclitrophicus* FF75 using the sspBCD-F/sspBCD-R primer pair (Table S3). The fragment was digested with KpnI and BamHI and ligated with pBluescript II SK(+), which had been digested using the same enzymes, generating pWHU730. The pWHU4393 to pWHU4395 plasmids, expressing SspA variants and SspBCD from *V. cyclitrophicus* FF75, were constructed using overlap extension PCR (OE-PCR). In terms of pWHU4393, two fragments, one of 877 bp and the other of 1,035 bp, both bearing the SspA_N150D_ mutation, were amplified from the genomic DNA of *V. cyclitrophicus* FF75 using the sspA-F/sspA-N150D-R and sspA-N150D-F/sspA-R primer pairs, respectively (Table S3). The two PCR products, sharing 17-bp overlapping ends, were combined and fused in a subsequent OE-PCR using the sspA-F/sspA-R primer pair. The resulting fusion product was digested with XbaI and SacII and ligated with pWHU730, which had been treated with the same enzymes to yield pWHU4393. Similarly, pWHU4394 and pWHU4395 were constructed with the sspA-C314S-F/sspA-C314S-R and sspA-R340E-F/sspA-R340E-R primer pairs, respectively (Table S3).

10.1128/mBio.00488-20.5 TABLE S3 Primer sequences used in this study. Download Table S3, PDF file, 0.01 MB. Copyright © 2020 Liu et al. 2020 Liu et al. This content is distributed under the terms of the Creative Commons Attribution 4.0 International license.

### Construction of pWHU4396 and pWHU4397.

A 1,633-bp (*dndA*) fragment was amplified from the genomic DNA of ZS-139 using the dndA-ZS139-F/dndA-ZS139-R primer pair (Table S3). The PCR fragment was ligated with pMMB67 that had been linearized by BamHI and SalI via an *in vitro* recombination method using the Hieff Clone Plus one-step cloning kit (Yeasen, China), generating pWHU4396. Similarly, a 1,557-bp fragment containing *sspA* from FF75 was cloned into pDSK519, generating pWHU4397.

### Construction of plasmids for protein purification.

A 1,089-bp (*sspA*) fragment was amplified from the genomic DNA of FF75 using the 28a-sspA-75-F/28a-sspA-75-R primer pair (Table S3), which was ligated with pET28a linearized by NdeI and XhoI using a recombination method *in vitro*, generating pWHU4388. pWHU4389, pWHU4391, and pWHU4392 were cloned in the same way using pWHU4394 and the genomic DNA of NCIMB400 as the PCR templates.

### Protein expression and purification.

The genes encoding the full-length SspA from *V. cyclitrophicus* FF75 as well as SspA and SspD from S. frigidimarina NCIMB400 were subcloned into the pET28a vector with N-terminal 6×His tags. The resulting plasmids were transformed into E. coli BL21(DE3) competent cells. Transformed cells were cultured at 37°C to an optical density at 600 nm (OD_600_) of 0.8, and protein expression was induced with 0.2 mM isopropyl-β-d-1-thiogalactopyranoside followed by incubation for 14 to 18 h at 16°C. After collection and resuspension, cells were disrupted using a cell homogenizer (JNBIO, Guangzhou, China). Cell debris was removed by centrifugation at 14,000 × *g* for 45 min at 4°C, and the supernatant was loaded onto a Ni^2+^-nitrilotriacetic acid (NTA) affinity chromatography column (GE Healthcare, Uppsala, Sweden). The eluted protein was further purified by size exclusion chromatography on a Superdex 200 gel filtration column (GE Healthcare, Uppsala, Sweden) in a buffer containing 10 mM Tris (pH 8.0), 100 mM NaCl, and 2 mM dithiothreitol. Peak fractions were pooled and concentrated to 10 mg/ml for crystallization. The C314S point mutant of the SspA protein was purified using the same procedure as that used for the wild-type SspA protein.

### Crystallization, data collection, and structure determination.

Crystals of SspA_C314S_ in complex with the cysteine substrate were grown at 14°C by the hanging-drop vapor-diffusion method with 1 μl of protein mixed with 1 μl of reservoir solution containing 1.8 M ammonium citrate (pH 7.0) (Hampton Research, USA). Crystals were cryoprotected in this crystallization buffer supplemented with 25% glycerol. The diffraction data were collected at the BL19U1 beamline at the National Center for Protein Sciences Shanghai (Shanghai, China) at 100 K. Diffraction data were processed using HKL3000 (42). The structure of SspA with a cysteine substrate was determined at 1.80 Å by the method of molecular replacement using the PHASER CCP4 program. The structure of Streptomyces lividans DndA (PDB code 3VAX) was utilized as the searching model after the mode-building by Coot and refinement by the REFMAC program of CCP4 (43, 44). The crystals belong to the *P*6_3_22 space group, and two molecules of the SspA-cysteine complexes were contained in each asymmetric unit. The final refined model had an *R*_work_/*R*_free_ of 16.58%/19.06%. The PROCHECK CCP4 program was used to evaluate the quality of the structure model, which indicated that the model exhibited good stereochemistry based on a Ramachandran plot.

### MD simulation.

The MD simulations were performed by the ff99Sbildn force field and AMBER 12 package (45, 46). From the X-ray crystal structure, the atomic coordinates of the SspA/Cys complex were obtained. The force field and AM1-bcc charges of the ligands were handled by the Antechamber module (47). System neutrality was maintained by adding counter-ions. The bonds involving hydrogen atoms were constrained by the SHAKE algorithm (48). A truncated octahedron box of TIP3P water models was applied in all systems with solvent layers 10 Å between the box edges and the solute surface (49). The long-range electrostatic interaction was evaluated by the partial mesh Ewald (PME) method (50). To relieve any structural clash, 1,000-step steepest descent minimization was used in the solvated system. Heating to 298 K and brief equilibrating for 20 ps in the NVT ensemble were performed with PMEMD of AMBER 12. Langevin dynamics with a time step of 2 fs were performed in the heating and equilibrating runs with friction constants of 1 ps. The simulation was performed at 298 K for nonspecific as well as specific systems with 100 ns for every system. CPPTRAJ was applied to process the trajectories (51), and the figure was plotted by OriginPro 9.1.

### NM analysis.

NM analysis was conducted as previously described (36). Briefly, the structural coordinates of SspA, DndA (PDB code 3VAX), and IscS (PDB code 4EB7) were submitted for NM analysis using the Elastic Network Model server (http://www.sciences.univ-nantes.fr/elnemo/start.html), which can compute the low-frequency normal modes of a protein. The first vibrational mode (i.e., the seventh NM with the lowest vibrational frequency is the most important vibrational mode) generated by the server was selected for further analysis.

### Molecular dynamics simulation for the SspA dimer.

The protein structure was obtained by X-ray crystallography. The protein molecule was centered in a cubic box sized 11.03 nm with the CHARMM 36 force field and TIP3P water model, applying GROMACS 2016.3 as the MD engine (52–58). The force field parameter of the PLP ligand was generated by the CHARMM General Force Field (CGenFF) (59). Systems were neutralized with 0.1 M NaCl. van der Waals interaction was truncated at 1.2 nm with the Lennard-Jones potential switched to zero gradually at 1.0 nm. Electrostatic interaction was evaluated by the PME method with a Coulomb cutoff of 1.2 nm (60). The LINCS algorithm was used to constrain bonds involving hydrogen atoms, allowing a time step of 2 fs (61). The system was first energy minimized using the steepest descent steps with a maximum force of 10.0 kJ · mol^−1^ · nm^−1^ and a maximum of 50,000 steps, and it was then equilibrated in the NVT ensemble at a temperature of 300 K for 300 ps and in the NPT ensemble at *P* = 10^5^ Pa for 20 ns. Temperature coupling and pressure coupling were performed leveraging the velocity-rescale and Parrinello-Rahman algorithm, respectively, with a coupling time of τ = 1 ps (62, 63). The MD simulation of NPT was conducted for 1 μs. The trajectories were recorded every 50 ps with 20,000 frames.

### SAXS data collection.

Synchrotron SAXS measurements in this study were performed at the BL19U2 beamline at the National Center for Protein Science Shanghai (Shanghai, China). The useful data, which were in the *q* range of 0.01 to 0.35 Å^−1^ (*q *= 4*π*sin*θ*/λ, 2*θ* is the scattering angle), were collected by a Pilatus detector. Measurement was conducted in a vacuum with an exposure time of 20 s in 21-s frames. The samples were monitored for possible radiation damage, and no radiation effects were detected. The X-ray wavelength was 1.03 Å. To remove aggregates and sediments, all samples were centrifuged at 12,000 × *g* for 20 min immediately prior to measurements. In the scattering curves, only the most informative part (between 0.01 Å^−1^ and 0.2 Å^−1^) could be used for structural analysis as a result of the considerable experimental noise at higher scattering angles. To determine the effects of concentration, samples containing six different concentrations (0.5, 1, 3, 5, 7, and 9 mg/ml) of SspA protein were prepared and measured. The cysteine substrate, which was present in a 10-fold (molar ratio)-greater concentration than SspA, was added directly before measurements to avoid air oxidation. During the measurements, there was no concentration dependence or aggregates observed.

### SAXS data processing.

The ATSAS program package was used to process all SAXS data (64). After subtracting the scattering of buffer from the signals of proteins, data were extrapolated to zero concentrations with standard procedures and the PRIMUS program (65). Both calculations and reconstructions used the resultant curves. Three-dimensional reconstructions of SspA were performed using DAMMIN and then compared with the SspA crystal structure by the SUPCOMB program (66, 67). The data set was fitted against predicted scattering profiles, which was calculated from atomic coordinates by the CRYSOL program (68).

### Clustering method.

Twenty thousand structures were clustered as described previously (37) based on the Cα-Cα distance similarity criterion as follows:

$$D\left(i,j\right)=\sqrt{\frac{1}{N_{2}}\underset{m,n}{\sum }{\left(d_{m,n}^{i}-d_{m,n}^{j}\right)}^{2}}$$

where $d_{m,n}^{i}$ is the Euclidean distance of the α-carbon atoms of residues *m* and *n* in structure *I* and *N*_2_ is the number of residue pairs.

Three clusters were determined from 20,000 structures with a cutoff of 1.5 Å. The average scattering profile of each cluster was then calculated by CRYSOL (69). The profilers were further regarded as the basis to fit the experimental scattering profile by minimizing the χ^2^ with 20,000 steps of Metropolis Monte Carlo as follows:

$${\text{χ}}^{2}=\frac{1}{L-1}\underset{s}{\sum }\frac{{\left[\sum_{i=1}^{N}P_{i}\times I_{i}\left(q_{s}\right)-I_{\text{exp}}\left(q_{s}\right)\right]}^{2}}{\sigma^{2}\left(q_{s}\right)}$$

where $\overset{N}{\underset{i=1}{\sum }}P_{i}\times I_{i}\left(q_{s}\right)$ is a sum over different structures for a defined wave vector, *q_S_*; the summation ∑*_S_* is performed over different *q* vectors; *L* is the number of *q* vectors; *I_i_*(*q*) is the averaged SAXS profile of each cluster; and *I*_exp_(*q_s_*) is the experimental SAXS profile. The populations of different clusters were obtained by fitting against the experimental SAXS profiles using a Monte Carlo procedure described previously (70).

### Molecular graphics.

All protein structure figures were generated by the PyMOL program (71).

### Data availability.

The atomic coordinates and structure factors of SspA-C314S in complex with the cysteine substrate have been deposited in the Protein Data Bank with the accession number 6M4J.

## References

[1] Mueller EG 2006 Trafficking in persulfides: delivering sulfur in biosynthetic pathways. Nat Chem Biol 2:185–194. doi:10.1038/nchembio779. 16547481

[2] Hidese R, Mihara H, Esaki N 2011 Bacterial cysteine desulfurases: versatile key players in biosynthetic pathways of sulfur-containing biofactors. Appl Microbiol Biotechnol 91:47–61. doi:10.1007/s00253-011-3336-x. 21603932

[3] Zheng L, White RH, Cash VL, Dean DR 1994 Mechanism for the desulfurization of L-cysteine catalyzed by the *nifS* gene product. Biochemistry 33:4714–4720. doi:10.1021/bi00181a031. 8161529

[4] Flint DH 1996 *Escherichia coli* contains a protein that is homologous in function and N-terminal sequence to the protein encoded by the *nifS* gene of* Azotobacter vinelandii* and that can participate in the synthesis of the Fe-S cluster of dihydroxy-acid dehydratase. J Biol Chem 271:16068–16074. 8663056

[5] Mihara H, Maeda M, Fujii T, Kurihara T, Hata Y, Esaki N 1999 A *nifS*-like gene, *csdB*, encodes an *Escherichia coli* counterpart of mammalian selenocysteine lyase. Gene cloning, purification, characterization and preliminary x-ray crystallographic studies. J Biol Chem 274:14768–14772. doi:10.1074/jbc.274.21.14768. 10329673

[6] Wang L, Chen S, Xu T, Taghizadeh K, Wishnok JS, Zhou X, You D, Deng Z, Dedon PC 2007 Phosphorothioation of DNA in bacteria by *dnd* genes. Nat Chem Biol 3:709–710. doi:10.1038/nchembio.2007.39. 17934475

[7] Wang L, Jiang S, Deng Z, Dedon PC, Chen S 2019 DNA phosphorothioate modification—a new multi-functional epigenetic system in bacteria. FEMS Microbiol Rev 43:109–122. doi:10.1093/femsre/fuy036. 30289455 PMC6435447

[8] Xiong L, Liu S, Chen S, Xiao Y, Zhu B, Gao Y, Zhang Y, Chen B, Luo J, Deng Z, Chen X, Wang L, Chen S 2019 A new type of DNA phosphorothioation-based antiviral system in archaea. Nat Commun 10:1688. doi:10.1038/s41467-019-09390-9. 30975999 PMC6459918

[9] Xiong X, Wu G, Wei Y, Liu L, Zhang Y, Su R, Jiang X, Li M, Gao H, Tian X, Zhang Y, Hu L, Chen S, Tang Y, Jiang S, Huang R, Li Z, Wang Y, Deng Z, Wang J, Dedon P, Chen S, Wang L 2020 SspABCD-SspE is a phosphorothioation-sensing bacterial defense system with broad antiphage activities. Nat Microbiol doi:10.1038/s41564-020-0700-6. 32251370

[10] Cao B, Chen C, DeMott MS, Cheng Q, Clark TA, Xiong X, Zheng X, Butty V, Levine SS, Yuan G, Boitano M, Luong K, Song Y, Zhou X, Deng Z, Turner SW, Korlach J, You D, Wang L, Chen S, Dedon PC 2014 Genomic mapping of phosphorothioates reveals partial modification of short consensus sequences. Nat Commun 5:3951. doi:10.1038/ncomms4951. 24899568 PMC4322921

[11] Chen C, Wang L, Chen S, Wu X, Gu M, Chen X, Jiang S, Wang Y, Deng Z, Dedon PC, Chen S 2017 Convergence of DNA methylation and phosphorothioation epigenetics in bacterial genomes. Proc Natl Acad Sci U S A 114:4501–4506. doi:10.1073/pnas.1702450114. 28400512 PMC5410841

[12] An X, Xiong W, Yang Y, Li F, Zhou X, Wang Z, Deng Z, Liang J 2012 A novel target of IscS in *Escherichia coli*: participating in DNA phosphorothioation. PLoS One 7:e51265. doi:10.1371/journal.pone.0051265. 23240007 PMC3519819

[13] You D, Wang L, Yao F, Zhou X, Deng Z 2007 A novel DNA modification by sulfur: DndA is a NifS-like cysteine desulfurase capable of assembling DndC as an iron-sulfur cluster protein in *Streptomyces lividans*. Biochemistry 46:6126–6133. doi:10.1021/bi602615k. 17469805

[14] Chen F, Zhang Z, Lin K, Qian T, Zhang Y, You D, He X, Wang Z, Liang J, Deng Z, Wu G 2012 Crystal structure of the cysteine desulfurase DndA from *Streptomyces lividans* which is involved in DNA phosphorothioation. PLoS One 7:e36635. doi:10.1371/journal.pone.0036635. 22570733 PMC3343029

[15] He W, Huang T, Tang Y, Liu Y, Wu X, Chen S, Chan W, Wang Y, Liu X, Chen S, Wang L 2015 Regulation of DNA phosphorothioate modification in *Salmonella enterica* by DndB. Sci Rep 5:12368. doi:10.1038/srep12368. 26190504 PMC4507180

[16] Xia S, Chen J, Liu L, Wei Y, Deng Z, Wang L, Chen S 2019 Tight control of genomic phosphorothioate modification by the ATP-modulated autoregulation and reusability of DndB. Mol Microbiol 111:938–950. doi:10.1111/mmi.14186. 30552823

[17] Yao F, Xu T, Zhou X, Deng Z, You D 2009 Functional analysis of *spfD* gene involved in DNA phosphorothioation in *Pseudomonas fluorescens* Pf0-1. FEBS Lett 583:729–733. doi:10.1016/j.febslet.2009.01.029. 19171139

[18] Hu W, Wang C, Liang J, Zhang T, Hu Z, Wang Z, Lan W, Li F, Wu H, Ding J, Wu G, Deng Z, Cao C 2012 Structural insights into DndE from *Escherichia coli* B7A involved in DNA phosphorothioation modification. Cell Res 22:1203–1206. doi:10.1038/cr.2012.66. 22525332 PMC3391021

[19] Xu T, Yao F, Zhou X, Deng Z, You D 2010 A novel host-specific restriction system associated with DNA backbone S-modification in *Salmonella*. Nucleic Acids Res 38:7133–7141. doi:10.1093/nar/gkq610. 20627870 PMC2978375

[20] Wang L, Chen S, Vergin KL, Giovannoni SJ, Chan SW, DeMott MS, Taghizadeh K, Cordero OX, Cutler M, Timberlake S, Alm EJ, Polz MF, Pinhassi J, Deng Z, Dedon PC 2011 DNA phosphorothioation is widespread and quantized in bacterial genomes. Proc Natl Acad Sci U S A 108:2963–2968. doi:10.1073/pnas.1017261108. 21285367 PMC3041111

[21] Kellner S, DeMott MS, Cheng CP, Russell BS, Cao B, You D, Dedon PC 2017 Oxidation of phosphorothioate DNA modifications leads to lethal genomic instability. Nat Chem Biol 13:888–894. doi:10.1038/nchembio.2407. 28604692 PMC5577368

[22] Tong T, Chen S, Wang L, Tang Y, Ryu JY, Jiang S, Wu X, Chen C, Luo J, Deng Z, Li Z, Lee SY, Chen S 2018 Occurrence, evolution, and functions of DNA phosphorothioate epigenetics in bacteria. Proc Natl Acad Sci U S A 115:E2988–E2996. doi:10.1073/pnas.1721916115. 29531068 PMC5879708

[23] Cupp-Vickery JR, Urbina H, Vickery LE 2003 Crystal structure of IscS, a cysteine desulfurase from *Escherichia coli*. J Mol Biol 330:1049–1059. doi:10.1016/s0022-2836(03)00690-9. 12860127

[24] Kaiser JT, Clausen T, Bourenkow GP, Bartunik HD, Steinbacher S, Huber R 2000 Crystal structure of a NifS-like protein from Thermotoga maritima: implications for iron sulphur cluster assembly. J Mol Biol 297:451–464. doi:10.1006/jmbi.2000.3581. 10715213

[25] Tirupati B, Vey JL, Drennan CL, Bollinger JM. Jr, 2004 Kinetic and structural characterization of Slr0077/SufS, the essential cysteine desulfurase from *Synechocystis* sp. PCC 6803. Biochemistry 43:12210–12219. doi:10.1021/bi0491447. 15379559

[26] Marinoni EN, de Oliveira JS, Nicolet Y, Raulfs EC, Amara P, Dean DR, Fontecilla-Camps JC 2012 (IscS-IscU)2 complex structures provide insights into Fe2S2 biogenesis and transfer. Angew Chem Int Ed Engl 51:5439–5442. doi:10.1002/anie.201201708. 22511353

[27] Shi R, Proteau A, Villarroya M, Moukadiri I, Zhang L, Trempe JF, Matte A, Armengod ME, Cygler M 2010 Structural basis for Fe-S cluster assembly and tRNA thiolation mediated by IscS protein-protein interactions. PLoS Biol 8:e1000354. doi:10.1371/journal.pbio.1000354. 20404999 PMC2854127

[28] Kim S, Park S 2013 Structural changes during cysteine desulfurase CsdA and sulfur acceptor CsdE interactions provide insight into the trans-persulfuration. J Biol Chem 288:27172–27180. doi:10.1074/jbc.M113.480277. 23913692 PMC3779715

[29] Behshad E, Bollinger JM Jr. 2009 Kinetic analysis of cysteine desulfurase CD0387 from *Synechocystis* sp. PCC 6803: formation of the persulfide intermediate. Biochemistry 48:12014–12023. doi:10.1021/bi802161u. 19883076

[30] Fujii T, Maeda M, Mihara H, Kurihara T, Esaki N, Hata Y 2000 Structure of a NifS homologue: X-ray structure analysis of CsdB, an *Escherichia coli* counterpart of mammalian selenocysteine lyase. Biochemistry 39:1263–1273. doi:10.1021/bi991732a. 10684605

[31] Lima CD 2002 Analysis of the *E. coli* NifS CsdB protein at 2.0 A reveals the structural basis for perselenide and persulfide intermediate formation. J Mol Biol 315:1199–1208. doi:10.1006/jmbi.2001.5308. 11827487

[32] Lundgren HK, Bjork GR 2006 Structural alterations of the cysteine desulfurase IscS of *Salmonella enterica* serovar Typhimurium reveal substrate specificity of IscS in tRNA thiolation. J Bacteriol 188:3052–3062. doi:10.1128/JB.188.8.3052-3062.2006. 16585765 PMC1447000

[33] Mihara H, Fujii T, Kato S, Kurihara T, Hata Y, Esaki N 2002 Structure of external aldimine of *Escherichia coli* CsdB, an IscS/NifS homolog: implications for its specificity toward selenocysteine. J Biochem 131:679–685. doi:10.1093/oxfordjournals.jbchem.a003151. 11983074

[34] Rybniker J, Pojer F, Marienhagen J, Kolly GS, Chen JM, van Gumpel E, Hartmann P, Cole ST 2014 The cysteine desulfurase IscS of *Mycobacterium tuberculosis* is involved in iron-sulfur cluster biogenesis and oxidative stress defence. Biochem J 459:467–478. doi:10.1042/BJ20130732. 24548275

[35] Gai Z, Wang Q, Yang C, Wang L, Deng W, Wu G 2016 Structural mechanism for the arginine sensing and regulation of CASTOR1 in the mTORC1 signaling pathway. Cell Discov 2:16051. doi:10.1038/celldisc.2016.51. 28066558 PMC5187391

[36] Zhang Y, Fu L, Qi X, Zhang Z, Xia Y, Jia J, Jiang J, Zhao Y, Wu G 2013 Structural insight into the mutual recognition and regulation between Suppressor of Fused and Gli/Ci. Nat Commun 4:2608. doi:10.1038/ncomms3608. 24217340 PMC5842692

[37] Daura X, Gademann K, Jaun B, Seebach D, van Gunsteren WF, Mark AE 1999 Peptide folding: when simulation meets experiment. Angew Chem Int Ed 38:236–240. doi:10.1002/(SICI)1521-3773(19990115)38:1/2<236::AID-ANIE236>3.0.CO;2-M.

[38] Kurihara T, Mihara H, Kato S, Yoshimura T, Esaki N 2003 Assembly of iron-sulfur clusters mediated by cysteine desulfurases, IscS, CsdB and CSD, from *Escherichia coli*. Biochim Biophys Acta 1647:303–309. doi:10.1016/s1570-9639(03)00078-5. 12686149

[39] Urbina HD, Silberg JJ, Hoff KG, Vickery LE 2001 Transfer of sulfur from IscS to IscU during Fe/S cluster assembly. J Biol Chem 276:44521–44526. doi:10.1074/jbc.M106907200. 11577100

[40] Xiong W, Zhao G, Yu H, He X 2015 Interactions of Dnd proteins involved in bacterial DNA phosphorothioate modification. Front Microbiol 6:1139. doi:10.3389/fmicb.2015.01139. 26539172 PMC4611135

[41] Shi M, Wosnick JH, Ho K, Keating A, Shoichet MS 2007 Immuno-polymeric nanoparticles by Diels-Alder chemistry. Angew Chem Int Ed Engl 46:6126–6131. doi:10.1002/anie.200701032. 17628481

[42] Otwinowski Z, Minor W 1997 Processing of X-ray diffraction data collected in oscillation mode. Methods Enzymol 276:307–326. doi:10.1016/S0076-6879(97)76066-X. 27754618

[43] Bailey S 1994 The CCP4 suite-programs for protein crystallography. Acta Crystallogr 50:760–763. 10.1107/S0907444994003112 15299374

[44] Emsley P, Cowtan K 2004 Coot: model-building tools for molecular graphics. Acta Crystallogr D Biol Crystallogr 60:2126–2132. doi:10.1107/S0907444904019158. 15572765

[45] Case D, Darden T, Cheatham T, Simmerling C, Wang J, Duke RE, Luo R, Walker RC, Zhang W, Merz K, Roberts B, Hayik S, Roitberg A, Seabra G, Swails J, Götz A, Kolossváry I, Wong KF, Paesani F, Kollman PA 2012 Amber 12. University of California, San Francisco, CA.

[46] Lindorff-Larsen K, Piana S, Palmo K, Maragakis P, Klepeis JL, Dror RO, Shaw DE 2010 Improved side-chain torsion potentials for the Amber ff99SB protein force field. Proteins 78:1950–1958. doi:10.1002/prot.22711. 20408171 PMC2970904

[47] Wang J, Wolf RM, Caldwell JW, Kollman PA, Case DA 2004 Development and testing of a general amber force field. J Comput Chem 25:1157–1174. doi:10.1002/jcc.20035. 15116359

[48] Ryckaert J-P, Ciccotti G, Berendsen H 1977 Numerical integration of the Cartesian equations of motion of a system with constraints: molecular dynamics of n-alkanes. J Comput Phys 23:327–341. doi:10.1016/0021-9991(77)90098-5.

[49] Jorgensen W, Chandrasekhar J, Madura J, Impey R, Klein M 1983 Comparison of simple potential functions for simulating liquid water. J Chem Phys 79:926–935. doi:10.1063/1.445869.

[50] Darden T, York D, Pedersen L 1993 Particle mesh Ewald: an Nlog (N) method for Ewald sums in large systems. J Chem Phys 98:10089–10092. doi:10.1063/1.464397.

[51] Roe DR, Cheatham TE III. 2013 PTRAJ and CPPTRAJ: software for processing and analysis of molecular dynamics trajectory data. J Chem Theory Comput 9:3084–3095. doi:10.1021/ct400341p. 26583988

[52] Huang J, MacKerell AD 2013 CHARMM36 all-atom additive protein force field: vValidation based on comparison to NMR data. J Comput Chem 34:2135–2145. doi:10.1002/jcc.23354. 23832629 PMC3800559

[53] Huang J, Rauscher S, Nawrocki G, Ran T, Feig M, de Groot BL, Grubmüller H, MacKerell AD 2017 CHARMM36m: an improved force field for folded and intrinsically disordered proteins. Nat Methods 14:71–73. doi:10.1038/nmeth.4067. 27819658 PMC5199616

[54] Best RB, Zhu X, Shim J, Lopes PEM, Mittal J, Feig M, MacKerell AD 2012 Optimization of the additive CHARMM all-atom protein force field targeting improved sampling of the backbone ϕ, ψ and side-chain χ1 and χ2 dihedral angles. J Chem Theory Comput 8:3257–3273. doi:10.1021/ct300400x. 23341755 PMC3549273

[55] Mackerell AD, Feig M, Brooks CL 2004 Extending the treatment of backbone energetics in protein force fields: Limitations of gas-phase quantum mechanics in reproducing protein conformational distributions in molecular dynamics simulations. J Comput Chem 25:1400–1415. doi:10.1002/jcc.20065. 15185334

[56] MacKerell AD, Bashford D, Bellott M, Dunbrack RL, Evanseck JD, Field MJ, Fischer S, Gao J, Guo H, Ha S, Joseph-McCarthy D, Kuchnir L, Kuczera K, Lau FTK, Mattos C, Michnick S, Ngo T, Nguyen DT, Prodhom B, Reiher WE, Roux B, Schlenkrich M, Smith JC, Stote R, Straub J, Watanabe M, Wiórkiewicz-Kuczera J, Yin D, Karplus M 1998 All-atom empirical potential for molecular modeling and dynamics studies of proteins. J Phys Chem B 102:3586–3616. doi:10.1021/jp973084f. 24889800

[57] Van Der Spoel D, Lindahl E, Hess B, Groenhof G, Mark AE, Berendsen H 2005 GROMACS: fast, flexible, and free. J Comput Chem 26:1701–1718. doi:10.1002/jcc.20291. 16211538

[58] Abraham MJ, Murtola T, Schulz R, Páll S, Smith JC, Hess B, Lindahl E 2015 GROMACS: hgh performance molecular simulations through multi-level parallelism from laptops to supercomputers. SoftwareX 1–2:19–25. doi:10.1016/j.softx.2015.06.001.

[59] Vanommeslaeghe K, Hatcher E, Acharya C, Kundu S, Zhong S, Shim J, Darian E, Guvench O, Lopes P, Vorobyov I, MacKerell AD 2010 CHARMM general force field (CGenFF): a force field for drug-like molecules compatible with the CHARMM all-atom additive biological force fields. J Comput Chem 31:671–690. doi:10.1002/jcc.21367. 19575467 PMC2888302

[60] Essmann U, Perera L, Berkowitz ML, Darden T, Lee H, Pedersen LG 1995 A smooth particle mesh Ewald method. J Chem Phys 103:8577–8593. doi:10.1063/1.470117.

[61] Hess B 2008 P-LINCS: a parallel linear constraint solver for molecular simulation. J Chem Theory Comput 4:116–122. doi:10.1021/ct700200b. 26619985

[62] Bussi G, Donadio D, Parrinello M 2007 Canonical sampling through velocity rescaling. J Chem Phys 126:14101. doi:10.1063/1.2408420. 17212484

[63] Melchionna S, Ciccotti G, Lee Holian B 1993 Hoover NPT dynamics for systems varying in shape and size. Mol Phys 78:533–544. doi:10.1080/00268979300100371.

[64] Konarev P, Petoukhov MV, Volkov V, Svergun DI 2006 ATSAS 2.1, a program package for small-angle scattering data analysis. J Appl Crystallogr 39:277–286. doi:10.1107/S0021889806004699. PMC4233345 25484842

[65] Konarev P, Volkov V, Sokolova A, Koch M, Svergun D 2003 PRIMUS: a Windows PC-based system for small-angle scattering data analysis. J Appl Crystallogr 36:1277–1282. doi:10.1107/S0021889803012779.

[66] Svergun DI 1999 Restoring low resolution structure of biological macromolecules from solution scattering using simulated annealing. Biophys J 76:2879–2886. doi:10.1016/S0006-3495(99)77443-6. 10354416 PMC1300260

[67] Kozin MB, Svergun DI, Embl B 2001 Automated matching of high- and low-resolution structural models. J Appl Crystallogr 34:33–41. doi:10.1107/S0021889800014126.

[68] Petoukhov M, Franke D, Shkumatov A, Tria G, Kikhney A, Gajda M, Gorba C, Mertens H, Konarev P, Svergun D 2012 New developments in the ATSAS program package for small-angle scattering data analysis. J Appl Crystallogr 45:342–350. doi:10.1107/S0021889812007662. 25484842 PMC4233345

[69] Svergun D, Barberato C, Koch MH 1995 CRYSOL—a program to evaluate X-ray solution scattering of biological macromolecules from atomic coordinates. J Appl Crystallogr 28:768–773. doi:10.1107/S0021889895007047.

[70] Tan P, Fu Z, Petridis L, Qian S, You D, Wei D-Q, Li J, Hong L 2019 A two-fold structural classification method for determining the accurate ensemble of protein structures. Commun Comput Phys 25:1010–1023. doi:10.4208/cicp.OA-2018-0140.

[71] Schrodinger, LLC. 2015 The PyMOL molecular graphics system, version 1.8. Schrodinger, LLC, New York, NY.

