# Supplementary material for: Structural Analysis of an l-Cysteine Desulfurase from an Ssp DNA Phosphorothioation System
Source: mBio. 2020 Apr 28;11(2):e00488-20. doi: 10.1128/mBio.00488-20 (PMC7188994; doi:10.1128/mBio.00488-20)
Supplement: TABLE S1 [file mBio.00488-20-st001.pdf]

TABLE S1 Data collection and refinement statistics

	SspA-C314S in complex cysteine
**Data collection**	
Space group	$P6_322$
Cell dimensions	
a, *b*, *c* (Å)	132.26, 132.26, 152.64
α, β, γ (°)	90, 90, 120
Resolution (Å)	50-1.52(1.57-1.52)
R_{merge}	10.3% (>100%)
I / σ_I	21.8(1.2)
$CC_{1/2}$	0.628
Completeness (%)	100.0 (100.0)
Redundancy	12.5 (10.3)
**Refinement**	
Resolution (Å)	50-1.80
No. of reflections	68,787
R_{work} / R_{free}	16.58/19.06
No. of atoms	
Protein	5,386
Cofactor	30
Substrate	14
Solvent	863
B factors (Å^2)	
Overall	22.16
Protein	20.55
Cofactor	17.86
Substrate	27.84
Solvent	32.26
RMSD bond length (Å)	0.0121
RMSD bond angles (°)	1.7572
Ramachandran plot	
Favored (%)	97.2
Allowed (%)	2.2
Disallowed (%)	0.6

$R_{merge} = \Sigma_h\Sigma_i |I_{h,i} – I_h|/\Sigma_h\Sigma_i I_{h,i}$ for the intensity (*I*) of observation i of reflection h. R factor $= \Sigma||F_{obs}| - |F_{calc}||/\Sigma|F_{obs}|$, where F_{obs} and F_{calc} are the observed and calculated structure factors, respectively. R_{free} = R factor calculated using 5% of the reflection data chosen randomly and omitted from the start of refinement. RMSD = root-mean-square deviation from ideal geometry. Data for the highest resolution shell are shown in parentheses.
