# Supplementary figures and images for: Structural Analysis of an l-Cysteine Desulfurase from an Ssp DNA Phosphorothioation System
Source: mBio. 2020 Apr 28;11(2):e00488-20. doi: 10.1128/mBio.00488-20 (PMC7188994; doi:10.1128/mBio.00488-20)

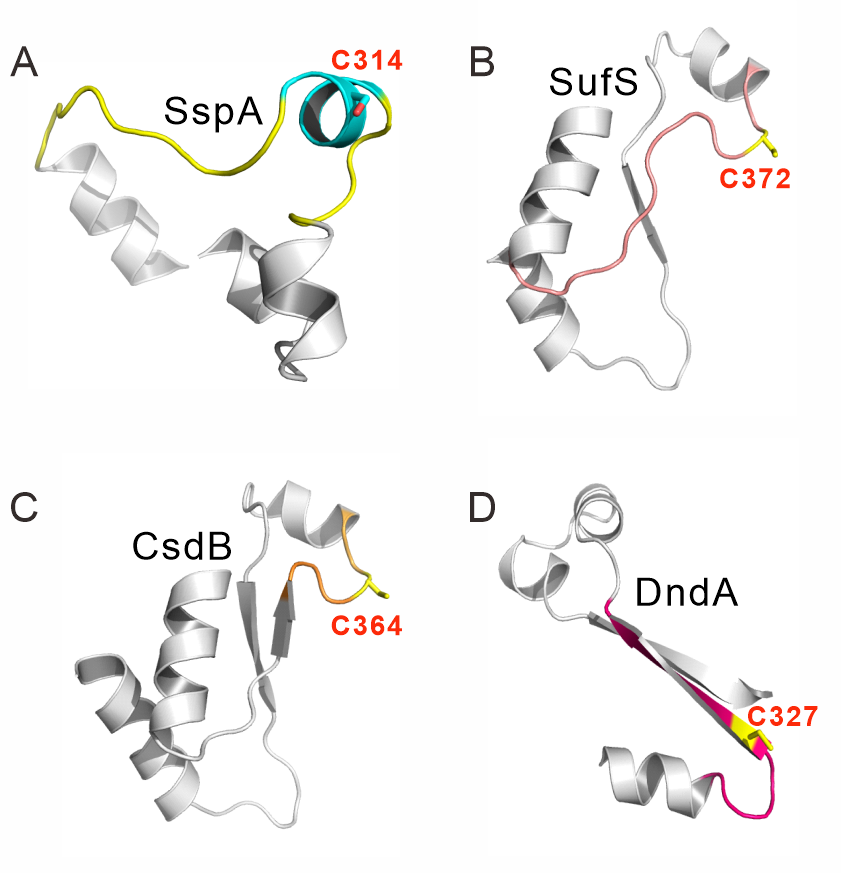

Supplement: FIG S1 [file mBio.00488-20-sf001.tif]

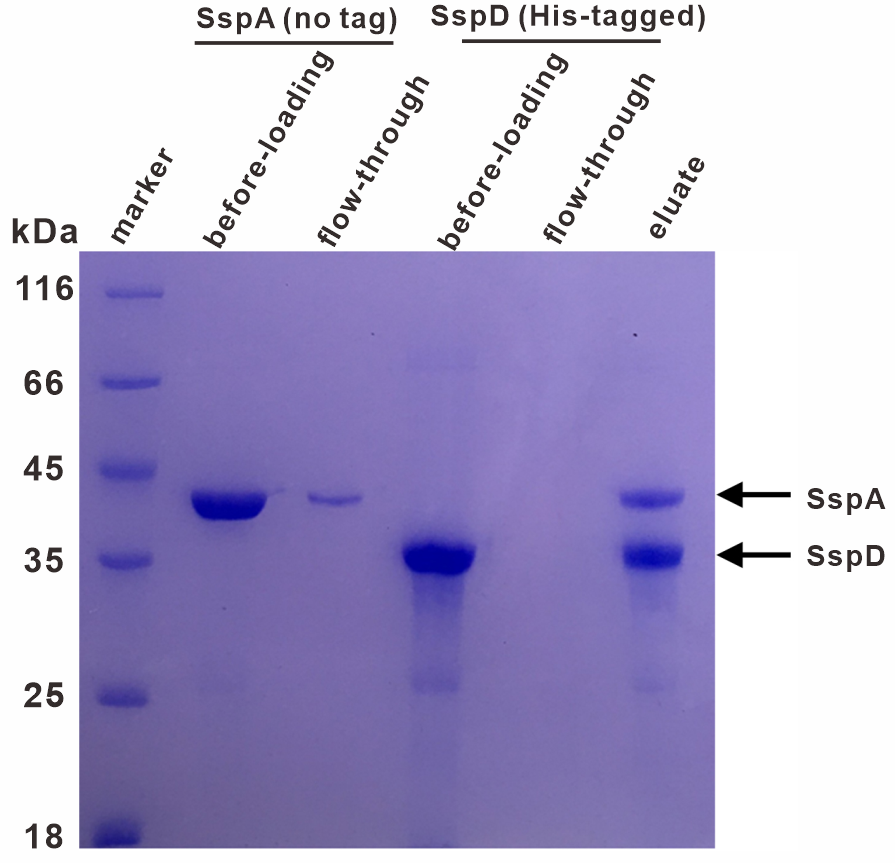

Supplement: FIG S2 [file mBio.00488-20-sf002.tif]
