# Supplementary material for: Structural Analysis of an l-Cysteine Desulfurase from an Ssp DNA Phosphorothioation System
Source: mBio. 2020 Apr 28;11(2):e00488-20. doi: 10.1128/mBio.00488-20 (PMC7188994; doi:10.1128/mBio.00488-20)
Supplement: TABLE S2 [file mBio.00488-20-st002.pdf]

TABLE S2 Strains and plasmids used in this study

Strains and plasmids	Characteristics	Source or reference
**Strains**		
V. cyclitrophicus FF75	d(C_{PS}C), GenBank: ATLT01000000	1
△*sspA*	*V. cyclitrophicus* FF75 derivative lacking *sspA*	2
Vibrio splendidus ZS-139	d(G_{PS}G), GenBank: NZ_AJZE00000000	1
Shewanella frigidimarina NCIMB400	d(C_{PS}C), GenBank: NC_008345 NZ_AAIV01000000 NZ_AAIV01000001-NZ_AAIV01000070	NCIMB
E. coli Trans1-T1	F^- φ80(*lacZ*)ΔM15 Δ*lac*X74 *hsdR*($r_K^-m_K^+$) Δ*recA1398endA1 tonA*	TransGen Biotech
E. coli BL21(DE3)	F^- *ompT gal dcm lon hsdS$_B$*($r_B^-m_B^-$) λ(DE3 [*lacI lacUV5*-T7p07 *ind*1 *sam*7 *nin*5]) [*mal*B^+]$_{K-12}$($λ^S$)	Novagen
**Plasmids**		
pBluescript II SK(+)	Cloning vector, 3 kb, Amp^r	3
pWHU730	pBluescript II SK(+) derivative with a 5.8-kb KpnI–BamHI fragment carrying *sspBCD* from *V. cyclitrophicus* FF75	This work
pWHU732	pBluescript II SK(+) derivative with a 5.8-kb KpnI–BamHI fragment carrying *sspBCD* and a 1.8-kb SacII-XbaI fragments carrying *sspA* from *V. cyclitrophicus* FF75, d(C_{PS}C)	2
pWHU4393	Derivative of pWHU732, expressing $SspA_{N150D}$BCD	This work
pWHU4394	Derivative of pWHU732, expressing $SspA_{C314S}$BCD	This work
pWHU4395	Derivative of pWHU732, expressing $SspA_{R340E}$BCD	This work
pMMB67	Cloning vector, 8.8 kb, Amp^r	4
pWHU4396	pMMB67 derivative carrying *dndA* from *Vibrio splendidus* ZS-139	This work
pDSK519	Cloning vector, 8.7 kb, Kan^r	5
pWHU4397	pDSK519 derivative carrying *sspA* from *V. cyclitrophicus* FF75	This work
pWHU4388	pET28a derivative expressing SspA from *V. cyclitrophicus* FF75, expression vector	This work
pWHU4389	pET28a derivative expressing $SspA_{C314S}$ from *V. cyclitrophicus* FF75, expression vector	This work
pWHU4391	pET28a derivative expressing SspA from *Shewanella frigidimarina* NCIMB400, expression vector	This work
pWHU4392	pET28a derivative expressing SspD from *Shewanella frigidimarina* NCIMB400, expression vector	This work

SI REFERENCES

1. Wang, L. et al. DNA phosphorothioation is widespread and quantized in bacterial genomes. *Proc Natl Acad Sci U S A* **108**, 2963-8 (2011).
2. Xiong, X. et al. SspABCD-SspE is a phosphorothioation-sensing bacterial defense system with broad antiphage activities. *Nature Microbiology* DOI: 10.1038/s41564-020-0700-6 (2020).
3. Alting-Mees, M. & Short, J. pBluescript II: Gene mapping vectors. *Nucleic acids research* **17**, 9494 (1989).
4. Fürste, J. et al. Molecular cloning of the plasmid RP4 primase in a multi host-range *tacP* expression vector. *Gene* **48**, 119-131 (1986).
5. Keen, N.T., Tamaki, S., Kobayashi, D. & Trollinger, D. Improved broad-host-range plasmids for DNA cloning in gram-negative bacteria. *Gene* **70**, 191-7 (1988).
