# Supplementary material for: Structural Analysis of an l-Cysteine Desulfurase from an Ssp DNA Phosphorothioation System
Source: mBio. 2020 Apr 28;11(2):e00488-20. doi: 10.1128/mBio.00488-20 (PMC7188994; doi:10.1128/mBio.00488-20)
Supplement: TABLE S3 [file mBio.00488-20-st003.pdf]

TABLE S3 Primers sequences used in this study

Primers	Sequences
sspA-F	GCTCTAGAGGAGAACATAATCGGTCTACCA
sspA-R	TCCCCGCGGCACTACTGCTGCGAGTCTAC
sspBCD-F	CGGGATCCAGCAGCTTGATACCATGAGATT
sspBCD-R	GGGGTACCTTGGAATGCCGATGTTAAGC
sspA-N150D-F	CTAGCTCATCGTTCACATGGTGAATTGT
sspA-N150D-R	TGTGAACGATGAGCTAGGAACGGTACAA
sspA-C314S-F	TAGCATTAGACGCCGAGCCTTGGGATAT
sspA-C314S-R	CTCGGCGTCTAATGCTATGTCTAACACA
sspA-R340E-F	AGCTAATTTCATATGTACGACGAGCTTC
sspA-R340E-R	TACATATGAAATTAGCTTACCTCCTTAT
dndA-ZS139-F	GAGCTCGGTACCCGGGGATCCTAATTGGCGATATCAGGAATAC
dndA-ZS139-R	CTTGCATGCCTGCAGGTCGACAGCAAAGCTCATTGGTTGTGATT
sspA-FF75-F	CGCACCTGCTTGATCGCGGCCGCACCAACAACTCACAAGATAGC
sspA-FF75-R	CAGGTCGACTCTAGAGGATCCTGAATAAGCACCTTTACATCC
28a-sspA-75-F	GTGCCGCGCGGCAGCCATATGATGACTAAGTATTTCGATTAC
28a-sspA-75-R	GTGGTGGTGGTGGTGCTCGAGCTACACTTTATAAGGAGGTAA
28a-sspA-400-F	GTGCCGCGCGGCAGCCATATGATGATCTATTTTGATACCGCA
28a-sspA-400-R	GTGGTGGTGGTGGTGCTCGAGCTATCTAGTCGCTTTTTTTACAG
28a-sspD-400-F	GTGCCGCGCGGCAGCCATATGATGACTGAAAAAGTTAAACATG
28a-sspD-400-R	GTGGTGGTGGTGGTGCTCGAGTTATTTATAACAGGTTACACAA
